# Utility of a slopes difference test for probing longitudinal multilevel aptitude treatment interactions: a simulation

**DOI:** 10.3389/fpsyg.2023.1156962

**Published:** 2023-06-27

**Authors:** Trey L. DeJong, Qi Chen

**Affiliations:** ^1^Department of Mathematics and Statistics, Center for Interdisciplinary Statistical Education and Research, Washington State University, Pullman, WA, United States; ^2^Department of Educational Psychology, The College of Education, University of North Texas, Denton, TX, United States

**Keywords:** aptitude-treatment interaction, skill-by-treatment interaction, slopes difference test, multilevel, longitudinal, precision education, simulation

## Abstract

To determine which interventions work best for which students, precision education researchers can examine aptitude-treatment interactions (ATI) or skill-by-treatment interactions (STI) using longitudinal multilevel modeling. Probing techniques like the slopes difference test fit an ATI or STI framework, but power for using slopes difference tests in longitudinal multilevel modeling is unknown. The current study used simulation to determine which design factors influence the power of slopes difference tests. Design factors included effect size, number of waves, number of clusters, participants per cluster, proportion of assignment to the treatment group, and intraclass correlation. Of these factors, effect size, number of waves, number of clusters, and participants per cluster were the strongest determinants of power, model convergence, and rates of singularity. Slopes difference tests had greater power in longitudinal multilevel modeling than where it is originally utilized: multiple regression.

## 1. Introduction

Conducting research featuring rigorous designs that effectively evaluate instructional interventions in the context of education is a difficult task. With experimentally strong designs historically avoided due to difficulty designing, executing, and funding (Gersten et al., 2000), many educational studies have been limited to correlational techniques that weaken the establishment of effective interventions for student outcomes (Villarreal et al., 2017; Johnson and Christensen, 2019). Lines of educational research that often use experimental or quasi-experimental designs to answer the question of which interventions work for which students (precision education; Cook et al., 2018), such as aptitude treatment interactions (ATIs; Cronbach and Snow, 1977) or skill-by-treatment interactions (STIs; Burns et al., 2010), are developing stronger and more cost-effective research designs. Preacher and Sterba (2019) give several recommendations for improving ATI research (e.g., using multiple repeated measure designs and ensuring appropriate power to test interactions), but techniques for answering research questions that follow these recommendations are often underutilized or even underdeveloped. For example, the slopes difference test developed by Dawson and Richter (2006) for use in multiple regression has a great conceptual fit for studying cross-level interactions in ATI or STI educational research, but to the best of the authors’ knowledge, no published research has utilized the slopes difference test in a multilevel context.

The current study develops a slopes difference test for the multilevel context and answers two sets of research questions. The primary focus of the study is to determine the power of using a slopes difference test: (1) what is the statistical power of the slopes difference test in a longitudinal multilevel analytical framework under different research design conditions? What factors affect this power and how? Additionally, if longitudinal multilevel models are likely to fail under certain design conditions, then researchers with limited resources or expectations of unfavorable design conditions should likely consider other designs when developing their research program. As such, the secondary focus of the current study is to determine under what kinds of designs the slopes difference test can be conducted: (2) what are the rates for convergence and singularity when fitting longitudinal multilevel models with the intention of conducting a slopes difference test under different research design conditions? What factors affect convergence and singularity rates and how?

Since the slopes difference test has not previously been utilized to examine ATIs/STIs—let alone interactions in a longitudinal multilevel context—it is important to review its development and utilization for the current study. While considering intervention research—ATIs and STIs in particular, longitudinal cluster-randomized trials, and techniques for interaction analysis (all reviewed in the subsequent sections), it becomes clear that the use of the slopes difference test in the areas of ATI and STI research is strongly supported. The following review defines precision education, reviews research design best suited to it, and provides evidence for the conceptual fit of the slopes difference test in a longitudinal multilevel analysis where an ATI/STI is being examined.

Intervention research is particularly important in the educational context (Ysseldyke et al., 2008). Since teachers have little to no control over many of the learning readiness factors of the students in their classrooms (Sullivan et al., 2004), teachers need to understand how best to approach students of varying backgrounds. In addition to practical experiences in the classroom, teachers can rely upon intervention research to determine whether various teaching approaches will be effective. Despite the obvious need for intervention research in education, until recently, empirical educational studies used rather rudimentary designs to determine the effectiveness of treatments. For example, in their content analysis of major school psychology journals from 2010 to 2014, Villarreal et al. (2017) found that only 11.1% of the articles included intervention methods in which an intervention was introduced and compared to a control group over time. The remaining designs were descriptive or correlational in nature. Being less powerful for determining or supporting causal relationships, these types of designs are less than ideal for drawing conclusions in educational research (Johnson and Christensen, 2019).

A more individualized approach to intervention research, called precision care, is a growing area across fields like medicine, psychology, and education. Precision care does not consider whether an intervention works or not, but rather which interventions work for whom and why (Cook et al., 2018). Referred to as *precision education* when used in school settings, this approach to intervention research does well to consider the social, emotional, academic, and physical health issues that impact students. Two frameworks that have been commonly used for precision education and related research, Aptitude-by-Treatment Interaction (ATI; Cronbach and Snow, 1977) and Skill-by-Treatment Interaction (STI; Burns et al., 2010), have been tested and debated for many years. As both are still being used for intervention research in education, it is necessary to review the background for both and consider their relevance to precision education.

An interaction effect may be defined as an effect among variables where the relationship between an independent variable (IV) and a dependent variable (DV) depends upon the levels of a second IV, called the moderator (Whisman and McClelland, 2005). In the context of an ATI, the relationship between the type or level of treatment applied and the desired outcome (e.g., academic score) depends upon the aptitude of the student toward the treatment—the moderator. Although, theoretically, aptitude or skill is generally considered to be the moderating variable for the relationship between treatment and the desired outcome, research tends to use treatment as the moderating variable in the analysis. Being a categorical variable, treatment is often easier to analyze as a moderator when analyzing an ATI or STI. Since it is mathematically arbitrary which variable is labeled as the independent variable or the moderator, researchers generally opt for the easier analysis by using treatment as the moderator (Preacher and Sterba, 2019). There is extensive literature discussing the distinctions between ATIs and STIs. However, due to limited space and the focus of the current study, researchers are encouraged to refer to other resources (e.g., Connor et al., 2004; Burns et al., 2018) for further details on their conceptual differences. Nevertheless, both ATI and STI are able to adopt a similar research design and analysis plan, so both are considered for the current study.

Several research design and statistical approaches have been created and adopted over the years to analyze these types of models. Thus, a review of previous research is helpful to learn about recommended designs. Among these, a longitudinal cluster-randomized trial fits well within the context of a precision education study aimed at examining an ATI or STI effect. Some related research designs and analyses are reviewed below to support the use of a longitudinal cluster-randomized trial in the current context.

Multilevel analysis, longitudinal design, and cluster-randomized trials (CRTs) have been used successfully to examine ATIs and STIs in educational research. For example, Hauk and Matlen (2016) utilized a CRT to determine the effectiveness of various types of web-based activities and testing systems for community college elementary algebra classes. Other studies like Connor et al. (2004) have utilized a longitudinal approach to examining STI effects. Still other studies examining ATIs or STIs in education have data that could be examined longitudinally, but use cross-sectional analyses instead (see Burns et al., 2018; Connor et al., 2018). Perhaps the most effective approach to addressing precision education questions in an ATI or STI framework is the longitudinal cluster-randomized trial (longitudinal-CRT), which covers a wide array of research design and analysis topics, including nested data structures and their analysis, longitudinal research design, and cluster-randomized trials (see Heo and Leon, 2009). Using such a design is beneficial for a number of reasons: (1) longitudinal analyses provide greater potential for an increase in statistical power which is crucial for examining interaction effects, (2) cluster randomization is easier to implement in an educational setting than other group assignment techniques, and (3) multilevel analyses respect the nested nature of data often found in longitudinal educational research data. We discuss the reason for these benefits and particular considerations for ATI/STI research in the following paragraphs.

Longitudinal research design has seen extensive use in ATI research. Aptitude growth design (Snow, 1991) examines pre- and post-treatment aptitude to see which treatments cause the greatest change for different pre-treatment aptitudes. Rogosa (1991) describes a growth curve analysis that can be used for this design that allows for a more statistically powerful analytical approach to ATI research. Because longitudinal designs measure the same participants repeatedly over a length of time, this means that more observations can be measured with a smaller number of participants.

Researchers often simplify their data from a longitudinal multiple-occasion design to a two-occasion format for analysis (see Burns et al., 2018; Connor et al., 2018). Preacher and Sterba (2019) reviewed several studies in which multilevel models measured only two occasions. Two-occasion designs are limited when analyzing an ATI/STI because a given ATI or STI may represent differences in how quickly students benefit from an intervention rather than a difference in the overall outcome (Smith and Sechrest, 1991). Since the goal of precision education is to impact individual outcomes for students, the chief concern of precision education research should be to show outcome differences for students of varying skills or aptitudes based on treatment. Using a multiple-occasion multilevel design – having at least three time points – solves this issue and allows for increased power to detect an ATI or STI (Preacher and Sterba, 2019).

To best understand how sample size, number of waves, or cluster size influences statistical power for longitudinal multilevel designs, it is important to understand how to assign participants to intervention groups. For ATI or STI educational research, therefore, the level at which participants are assigned to treatment groups should be considered.

When assigning students to treatment or control groups, a number of options are available. The most obvious is to randomly assign individual students to treatment or control groups. For large-scale interventions, however, cluster-randomized trials (CRTs) are considered the gold standard (Campbell et al., 2000). For CRTs, participants belong to clusters (i.e., classrooms), and are assigned to treatment or control by clusters. In education, where students often belong to classrooms, it can be difficult to assign participants to groups that are different from already existing classrooms. Since teachers serve as a natural and useful way to introduce an intervention, assigning whole classrooms rather than individual students to groups into an intervention means that extra steps are not needed in order to introduce interventions to students assigned to different groups. CRTs, being uniquely suitable for interventions where individuals naturally belong to nested data structures, work well for educational research (Glaman et al., 2020) including ATI research (Preacher and Sterba, 2019).

With increased statistical power available to longitudinal-CRT researchers (Kwok et al., 2008), commonly underpowered statistical tests, such as those used to discover and probe interaction effects, have a greater chance of arriving at statistically significant results. Interactions effects—as defined earlier in this review—are the phenomena of interest when researching an ATI or STI. In the following, therefore, we review different techniques for examining interactions and define probing techniques that are of interest to ATI or STI researchers.

To determine if an ATI or STI is meaningful, interactions must be analyzed using statistical analysis. While more general techniques are commonly known for examining interactions (i.e., an omnibus interaction), a greater depth of understanding can be gained by utilizing probing techniques on an interaction to better understand the precise relationships between each of the variables. We review below both conceptually and mathematically how interactions are tested starting with an overall test and working toward the probing technique of interest for the current study: the slopes difference test.

Interaction effects are commonly examined using an omnibus analysis to inform the researcher of the existence of a moderator (Durand, 2013). Consider a regression equation where there is one interaction term:

(1a) $$y_{i}=\beta_{0}+\beta_{1}x_{i}+\beta_{2}z_{i}+\beta_{3}x_{i}z_{i}+\varepsilon_{i}$$

where the *β_s_* represent regression weights and x and z represent the independent variable and moderator, respectively. The estimated value for *β*_3_ represents the weight of the omnibus interaction. This coefficient can be tested for statistical significance by comparing the critical ratio—defined below in Equation (1b)—to a *t*-distribution:

(1b) $$t=\frac{{\hat{\beta }}_{3}}{{\mathrm{SE}}_{{\hat{\beta }}_{3}}}.$$

While the above omnibus technique informs the researcher of the existence of an interaction, the interaction can be further examined using probing techniques such as the simple slopes technique (Aiken and West, 1991), the Johnson-Neyman technique (Johnson and Neyman, 1936), and the slopes difference test (Dawson and Richter, 2006). Precision education aims to understand which treatments work *best* for which students, not just which treatments work for which students. Of the three types of probing techniques mentioned here, the slopes difference test is the most informative and would be fit for probing an interaction effect in precision education research. The slopes difference test specifies levels of the moderator for which to determine the relationship between the predictor and outcome (i.e., the slopes relating predictor to outcome) and then tests for statistical significance *between* these slopes. In order to better understand how interactions are analyzed and probed, it is necessary to review the simple slopes technique, which provides the analytical foundation for the slopes difference test.

Equation (1a) can be reorganized to reflect a simple intercept and simple slope:

(1c) $$y_{i}=\left(\beta_{0}+\beta_{2}z_{i}\right)+\left(\beta_{1}+\beta_{3}z_{i}\right)x_{i}+\varepsilon_{i}$$

where simple intercept, $\beta_{0}+\beta_{2}z_{i}$ , is denoted as ${\hat{\omega }}_{0}$ and simple slope, $\beta_{1}+\beta_{3}z_{i}$ , is denoted as ${\hat{\omega }}_{1}$ , and both ${\hat{\omega }}_{0}$ and ${\hat{\omega }}_{1}$ are considered to be compound coefficients (Preacher et al., 2006).

Like the critical ratio for an omnibus significance test, the critical ratio for either the simple intercept or the simple slope can be calculated and compared to a *t*-distribution with df=N−p−1 , where *N* is the sample size and *p* is the number of independent variables, to test for statistical significance. The critical ratio for the simple slopes technique, therefore, is the following:

(1d) $$t=\frac{{\hat{\omega }}_{1}}{{\mathrm{SE}}_{{\hat{\omega }}_{1}}}.$$

This critical ratio changes depending on what level of the moderator is specified and is used to test a null hypothesis that t=0 . Therefore, the critical ratio for simple slope tells a researcher whether the relationship between the IV and the DV is statistically significantly different from zero for each level of treatment (Preacher et al., 2006).

Somewhat more nuanced, the slopes difference test tells a researcher whether the simple slopes for each of the treatment groups have statistically significant differences from one another (Dawson and Richter, 2006). This becomes particularly useful when there are several simple slopes to calculate, such as a three-way interaction scenario. Consider a general equation for a three-way interaction:

(1e) $$\begin{array}{l} y_{i}=\beta_{0}+\beta_{1}x_{i}+\beta_{2}z_{i}+\beta_{3}w_{i}+\beta_{4}x_{i}z_{i}\\ \;\;\;\;\;\;+\beta_{5}x_{i}w_{i}+\beta_{6}w_{i}z_{i}+\beta_{7}x_{i}z_{i}w_{i}+\varepsilon_{i} \end{array}$$

where *z* and *w* are the moderators. The values in the equation can again be reorganized to group simple intercept and simple slope terms together:

(1f), $$\begin{array}{l} y_{i}=(\beta_{0}+\beta_{2}z_{i}+\beta_{3}w_{i}+\beta_{6}w_{i}z_{i})\;\\ \;\;\;\;\;\;\;+(\beta_{1}+\beta_{4}z_{i}+\beta_{5}w_{i}+\beta_{7}z_{i}w_{i})x_{i}+\varepsilon_{i} \end{array}$$

and conditional values of the moderators, *z* and *w*, may be chosen. If high and low values of both moderators are chosen as the conditional values, then four simple slopes would exist: $z_{H}\;\mathrm{with}\;w_{H}$ , $z_{L}\;\mathrm{with}\;w_{H}$ , $z_{H}\;\mathrm{with}\;w_{L}$ , with $z_{L}\;\mathrm{with}\;w_{L}$ . The slopes difference test yields more information for researchers about how interactions change across levels of the moderators (Dawson and Richter, 2006).

As interactions become more complex, such as situations where a cross-level interaction is examined, probing techniques like the slopes difference test become crucial for understanding them. A cross-level interaction refers to situations where a higher-level moderator (e.g., aptitude or treatment) is considered to influence the nature or strength of the relationship between two lower-level variables (e.g., time predicting a student achievement outcome; Aguinis et al., 2013). Since three-way cross-level interactions naturally happen in longitudinal-CRT models that contain more than two levels (Raudenbush and Bryk, 2002), the slopes difference technique enables researchers to probe interactions in ATI/STI research where assignment to treatment and student aptitudes/skills moderate growth in achievement outcomes. ATI or STI effects may be missed or misrepresented without using probing techniques (Preacher et al., 2006).

Although the simple slopes technique has been extended from linear regression to longitudinal multilevel models (Preacher et al., 2006), the slopes difference test has not been extended to these types of models. Dawson and Richter (2006) utilized Monte Carlo simulation within a traditional multiple-regression context to determine the types of conditions under which slopes difference tests might be considered powerful enough to be practically useful. Considering the conceptual fit of slopes difference tests for cross-level interactions in longitudinal multilevel models, the practical utility of these models in the context of precision education must be determined. It was the purpose of the present study to extend the slopes difference test to a longitudinal multilevel context and determine its utility with conditions commonly found in precision education research.

When attempting to fit a complex statistical model, there are two warnings that may appear: non-convergence of the model and singularity of the model. When a non-convergence warning appears, the analysis fails to produce any results (i.e., estimated parameters or model summary). A singularity warning means that although the model was still able to produce results, the estimated parameters and model fit indices are untrustworthy. Although these warnings are not the primary focus of the current study, their presence in an analysis would make it difficult or impossible to utilize the slopes difference test in a multilevel model. Interpreting models with singular or non-convergent warnings is at best difficult and at worst not recommended (Linck and Cunnings, 2015). If a particular research design tends to lead to singularity or non-convergence, then researchers should avoid those research designs. As such, rates of non-convergence and singularity must be considered to inform researchers of whether these issues are likely to occur for their data.

## 2. Materials and methods

The goal of the present study was to determine the utility of the slopes difference test for a longitudinal multilevel model to probe an ATI or STI effect. Specifically, the following sets of research questions were asked:

1. What is the statistical power of the slopes difference test in a longitudinal multilevel analytical framework under different research design conditions? What factors affect this power and how?
2. What are the rates for convergence and singularity when fitting longitudinal multilevel models with the intention of conducting a slopes difference test under different research design conditions? What factors affect convergence and singularity rates and how?

Dawson and Richter (2006) extended the statistical test for simple slopes to determine if simple slopes from a three-way interaction are statistically significantly different from each other. Following the example of Dawson and Richter (2006), a Monte Carlo simulation was conducted to examine the statistical power of the slopes difference test across varying design conditions in a longitudinal multilevel context. Power was defined as the probability of finding a statistically significant effect when it should, in fact, exist (Cohen, 1988). In the current context, power was represented by the number of times a slope difference was found for a randomly drawn sample from a simulated population with specified parameters when a slope difference did exist in that population. Following recommendations from previous research (Dawson and Richter, 2006; Preacher and Sterba, 2019), we first established the method for using slopes difference tests in ATI/STI research, and then examined power across research design conditions. Once power across these conditions was determined, we investigated how these factors impacted power for the slopes difference test.

### 2.1. Simulation

With the research question and model in mind, a simulation was conducted using a Monte Carlo simulation in *R Studio* (RStudio Team, 2015). In this type of study, population values are created that align with specified data conditions and then analyzed to answer research questions related to the statistical analysis and/or parameter estimates of interest (see Dawson and Richter, 2006, for a similar study and process). Thus, parameters in the model had to be determined so that population values could be simulated. The following sections summarize the model, specify model parameters, and determine conditions that help to answer the research questions.

#### 2.1.1. The data generation model

Following the recommendation by Preacher and Sterba (2019) to use a model that is as parsimonious as possible and by utilizing a modified version of a three-level longitudinal model (Chen et al., 2010), the following longitudinal multilevel model was created:

- Level 1 (Occasion)

(2a) $$Y_{tij}=\pi_{0ij}+\pi_{1ij}\left({\mathrm{Time}}_{tij}\right)+e_{tij}$$

(2b) With etij~N(0,σ2)

- Level 2 (Student)

(2c) $$\pi_{0ij}=\beta_{00j}+\beta_{01j}\left(\mathrm{Aptitude}/{\mathrm{Skill}}_{ij}\right)+r_{0ij}$$

(2d) $$\pi_{1ij}=\beta_{10j}+\beta_{11j}\left(\mathrm{Aptitude}/{\mathrm{Skill}}_{ij}\right)+r_{1ij}$$

(2e) $$\mathrm{With}\left[\begin{array}{c} r_{0ij}\\ r_{1ij} \end{array}\right]\sim \mathrm{MVN}\left(\left[\begin{array}{c} 0\\ 0 \end{array}\right],\left[\begin{array}{cc} \tau_{\pi 00} & \tau_{\pi 01}\\ \tau_{\pi 10} & \tau_{\pi 11} \end{array}\right]\right)$$

- Level 3 (Classroom)

(2f) $$\beta_{00j}=\gamma_{000}+\gamma_{001}\left({\mathrm{Treatment}}_{j}\right)+\mu_{00j}$$

(2g) $$\beta_{01j}=\gamma_{010}+\gamma_{011}\left({\mathrm{Treatment}}_{j}\right)$$

(2h) $$\beta_{10j}=\gamma_{100}+\gamma_{101}\left({\mathrm{Treatment}}_{j}\right)$$

(2i) $$\beta_{11j}=\gamma_{110}+\gamma_{111}\left({\mathrm{Treatment}}_{j}\right)$$

(2j) $$\mathrm{With}\mu_{00j}\sim N\left(0,\tau_{\beta_{00}}\right)$$

where *Aptitude/Skill* is a continuous variable and *Treatment* is a dichotomous variable, with 0 representing a control group and 1 representing an intervention group. The combined/mixed model is as follows:

(2k) $$\begin{array}{l} Y_{tij}=\gamma_{000}+\gamma_{001}{\left(\mathrm{Treatment}\right)}_{j}+\gamma_{010}\left(\mathrm{Aptitude}\right)+\gamma_{100}{\left(Time\right)}_{tij}\\ +\gamma_{011}{\left(\mathrm{Treatment}\right)}_{j}\left(\mathrm{Aptitude}\right)+\gamma_{101}{\left(\mathrm{Treatment}\right)}_{j}{\left(\mathrm{Time}\right)}_{tij}\\ +\gamma_{110}\left(\mathrm{Aptitude}\right){\left(\mathrm{Time}\right)}_{tij}+\gamma_{111}{\left(\mathrm{Treatment}\right)}_{j}\left(\mathrm{Aptitude}\right){\left(\mathrm{Time}\right)}_{tij}\\ +r_{1\mathrm{ij}}{\left(\mathrm{Time}\right)}_{tij}+e_{tij}+r_{0\mathrm{ij}}+\mu_{00j}\; \end{array}$$

This model fits the context of a cluster-randomized trial (CRT) where assignment to a treatment is done at the classroom level. Only two treatment groups were chosen to ensure use of the simplest form of a model for this research context. In accordance with Preacher and Sterba’s (2019) observation that many ATI research articles do not expect the relationship between aptitude – or skills – and achievement to vary across clusters, Equations (2g)–(2i) constrain $\beta_{01j}$ , $\beta_{10j}$ , and $\beta_{11j}$ to vary across treatments but not across classrooms. Once the framework for the statistical model was established, parameters for simulation conditions needed to be specified. The achievement (outcome) variable was simulated by saving fitted values from the longitudinal multilevel model with the following fixed parameters and random effects as described and specified in the variance–covariance matrix below. Details regarding variable scaling and model variability introduced by the random effects of the model are described in Section 2.1.2.

#### 2.1.2. Fixed-value parameters

Eight fixed-effect coefficients (i.e., $\gamma_{000}$ , $\gamma_{001}$ , $\gamma_{010}$ , $\gamma_{011}$ , $\gamma_{100}$ , $\gamma_{101}$ , $\gamma_{110}$ , and $\gamma_{111}$ ) and five variances and covariance of the random effects (i.e., $\sigma^{2}$ , $\tau_{\pi 00}$ , $\tau_{\pi 01}$ , $\tau_{\pi 11}$ , and $\tau_{\beta 00}$ ) had to be specified. Using plausible values that fit with precision education research and the proposed model, fixed gamma values were specified ( $\gamma_{000}=0$ ; $\gamma_{001}=0.1$ ; $\gamma_{010}=0.5$ ; $\gamma_{011}=0.3$ ; $\gamma_{100}=0.1$ ; $\gamma_{110}=0.5$ ; $\gamma_{111}=0.1$ ). For the purposes of the simulation, we used a scale similar to that of centered IQ, with a mean of 0 and a standard deviation of 15, consistent with scales used in Connor et al. (2018) for both the outcome variable, achievement, and the aptitude variable. Therefore, aptitude values were randomly sampled from a distribution with a mean of 0 and standard deviation of 15. Aligning with a precision education research scenario where treatments may be targeting students of lower aptitudes, we determined the following: Treatment would appear to have only a small main effect, indicating that the treatment is only somewhat effective, aptitude would have a strong effect suggesting a strong relationship between a student’s aptitude and his/her achievement, time would have only a moderate effect to indicate that a student’s achievement grows over time, the interaction between aptitude and time would have a strong effect, meaning that achievement growth strongly depends on a student’s aptitude, the interaction between treatment and aptitude would have a small effect suggesting that the effect of a treatment on achievement changes little across aptitudes, and the interaction between treatment, aptitude, and time would have a small effect (implying that there is only a small moderating effect of aptitude and treatment on achievement growth) for consistency. Values used for small (0.1), medium (0.3), and large (0.5) effects are consistent with Cohen’s (1988) rules of thumb. The coefficient for the interaction between treatment and time, $\gamma_{101}$ , is relevant for determining the effects size for the slopes difference test. Therefore, its value will be discussed and specified in the next section.

In addition to gamma values, some variances and covariance of the random effects were fixed and specified according to Raudenbush and Liu’s (2001) medium effect size criteria as:

$$\sigma^{2}=1.0,T_{\pi }=\left[\begin{array}{cc} \tau_{\pi 00} & \tau_{\pi 01}\\ \tau_{\pi 10} & \tau_{\pi 11} \end{array}\right]=\left[\begin{array}{cc} 0.200 & 0.050\\ 0.050 & 0.100 \end{array}\right]$$

These values were fixed across all conditions of the simulation. Variability for the achievement variable was introduced via the random effects at each level in Equations (2b, 2e, 2j) (i.e., $e_{tij},r_{0ij},r_{1ij},and\;u_{00j}$ ). The variance for the classroom level, $\tau_{\beta 00}$ , will vary in accordance with the ICC conditions set for the simulation and is, therefore, discussed in the next section. In the following, we discuss and specify the design conditions set for the simulation.

#### 2.1.3. Simulation conditions

##### 2.1.3.1. Effect size

The slopes difference test can be used to compare any two pairs of slopes. If only high and low aptitudes are selected as conditional values and there are two treatment conditions (four unique slopes), six pairs of slopes can be compared. In order to test the effectiveness of the slopes difference test across the conditions to be specified for this simulation, it is only necessary to examine one of the pairs of slopes for a difference. Statistically, it does not matter which pair of slopes is compared because the same approach is used for all slope comparisons. For example, Dawson and Richter (2006) only examined one slope difference in their simulation.

In the context of ATI or STI, it makes the most sense to examine slopes where the first moderator, aptitude/skill, is constrained to a value—low for our purposes—and the slopes for both zero and one values in the second moderator, treatment, are compared (i.e., compare “Aptitude_Low_ and Control” with “Aptitude_Low_ and Treatment”). Therefore, the standardized version of formula *d* from Dawson and Richter (2006) was adjusted to calculate a *t*-value and test for statistical significance:

(3a) $$t=\frac{\gamma_{101}-\gamma_{111}}{\sqrt{{\mathrm{var}}_{\gamma 101}+{\mathrm{var}}_{\gamma 111}+2{\mathrm{cov}}_{\gamma 101\gamma 111}}}$$

The numerator in Equation (3a) represents the effect of the treatment condition on the relationship between time and achievement for a low ( z=−1 ) conditional value of aptitude/skill (i.e., the difference between the treatment and control group over time for low-aptitude students). Additionally, the fixed effect coefficient $\gamma_{101}$ was determined by the condition of the effect size for the simulation. The value of $\gamma_{101}$ was set as 0.6, 0.4, and 0.2 to represent a large (0.5), medium (0.3), and small (0.1) effect size conditions, respectively, for the slopes difference test.

##### 2.1.3.2. Intraclass correlation

The value of the intraclass correlation (ICC) was also specified to vary across three conditions: two that were chosen as real-world values, small and medium, from Hedges and Hedberg (2007) and one that represented a larger plausible value. Hedges and Hedberg (2007) found that depending on the type of achievement and the achievement level of the school (average or low achievement), the ICC values differed. Therefore, we utilized an ICC reflecting average achieving students (0.086) and one reflecting low-achieving students (0.113). A third ICC, 0.2, was also included to ensure that larger levels of ICC that are commonly found in educational research were considered (Chen et al., 2010). From these ICCs, $\tau_{\beta 00}$ was calculated based on the equation $\mathrm{ICC}=\tau_{\beta 00}/\left(\sigma^{2}+\tau_{\pi 00}+\tau_{\beta 00}\right)$ (Raudenbush and Bryk, 2002). For the smaller ICC (0.086) $\tau_{\beta 00}=0.113$ , for the moderate ICC (0.113) $\tau_{\beta 00}=0.153$ , and for the large ICC (0.2) $\tau_{\beta 00}=0.3$ . As such, these represent three ICC conditions for simulation.

##### 2.1.3.3. Sample and cluster size

Sample sizes for all three levels of the model were also varied. For the first level, the number of waves was varied across three conditions—three, four, and five—where the waves are considered to be equally spaced apart in time. Although Chen et al. (2010) used only four waves, we also considered other conditions because tests for interaction effects are often underpowered (Durand, 2013). This extra sensitivity to power could have led to the slopes difference test being especially sensitive to the number of waves in the study. After centering and spacing the waves by one, the values for the wave conditions were the following for three, four, and five wave conditions, respectively: −1, 0, and 1; −1.5, −0.5, 0.5, and 1.5; and − 2, −1, 0, 1, and 2.

Chen et al. (2010) conducted a comprehensive literature review of the education literature to determine the size and number of clusters (sample size for Levels 2 and 3, respectively). They settled upon using 20 and 40 cases per cluster and on 30, 50, and 80 as the number of clusters. After adjusting for more typical classroom sizes in the United States, the conditions for cases per cluster in the current study were determined to be 10, 20, and 30. Additionally, to increase the number of conditions for the number of clusters (i.e., the number of classrooms in the study) and limit the number of classrooms to what is typical in research studies, the conditions for the number of clusters were set to be 20, 30, 40, and 50.

Also relevant to the sizes of these clusters is the proportion of assignment to the treatment group at the school level. Stone-Romero et al. (1994) discussed the effects of categorical variables on power and recommended considering the proportion of participants belonging to each category. Treatment was a dichotomous variable in this simulation, so two levels of proportions for assignment to the treatment group: 0.5 and 0.3 (Stone-Romero et al., 1994) helped to determine whether different proportions of assignment to the treatment group affected statistical power.

#### 2.1.4. Analysis

The proposed simulation used a 3 (number of waves: three, four, or five) × 3 (students per classroom: 10, 20, or 30) × 4 (number of classrooms: 20, 30, 40, or 50) × 2 (proportions assigned to treatment group: 0.5 or 0.3) × 3 (ICC: 0.113, 0.086, or 0.2) × 3 (effect size for the slope difference: 0.1, 0.3, or 0.5) factorial design to simulate the data, resulting in 648 unique conditions. The simulation included 1,000 replications for each condition, yielding a total of 648,000 datasets. Once the data were simulated and saved using *R Studio* (RStudio Team, 2015), the accuracy of the parameter estimates was examined.

After parameter estimates were deemed accurate, three outcomes were examined: power, non-convergence, and singularity. Power, the probability of finding a statistically significant result when an effect does in-fact exist, is a useful outcome for determining whether the slopes difference test is likely to find an effect. Power for each condition was determined by finding the proportion of statistically significant results across all replications under each condition. To answer the second set of research questions, non-convergence rates and singularity rates were determined as the proportion of non-convergence and the proportion of singularity across all replications under each condition.

An ANOVA was used to determine how design factors and their interactions influenced power, non-convergence rates, and singularity rates for the slopes difference test. In addition to examining statistical significance, measures of effect size are crucial for determining the importance of effects in an ANOVA (Cohen, 1994). Therefore, effect sizes for the conditions, $\eta^{2}\;\left(i.e.,\eta^{2}={\mathrm{SS}}_{\mathrm{Effect}}/{\mathrm{SS}}_{\mathrm{Total}}\right)$ , were calculated for significant effects to determine which effects were the most meaningful.

## 3. Results

Data consisting of 1,000 replications across 648 unique conditions were simulated. Outcomes for data analysis included power per design condition across all replications, as well as convergence rate and singularity rate for supplementary analyses per design condition across all replications. Figures, ANOVA results, and *post-hoc* results are shown for power, convergence, and singularity. *Post-hoc* tests were only conducted for factors where $\eta^{2}>0.03$ to avoid interpreting factors that only explain a small portion of variance. For all statistical tests, α=0.05 . Results from all simulation conditions are also available via the following link: https://osf.io/rp23n/?view_only=e326f41934d8456ca1b8bc4ea8bf3da3.

### 3.1. Power for the slopes difference test across simulation conditions

Relevant to both research questions, power across all replications was examined first with descriptive statistics, and secondly with an ANOVA including both main and interaction effects. Effect sizes ( $\eta^{2}$ ) and Tukey’s HSD were then calculated to assist the interpretation of results. Tukey’s HSD is a *post-hoc* test that falls between the more conservative *Bonferroni method* and the more liberal *Fisher’s LSD* and allows for ANOVA results to be broken down into pairwise comparisons while also controlling for the inflation of the family-wise error rate (Abdi and Williams, 2010).

#### 3.1.1. Descriptive statistics for power

**Figure 1:**
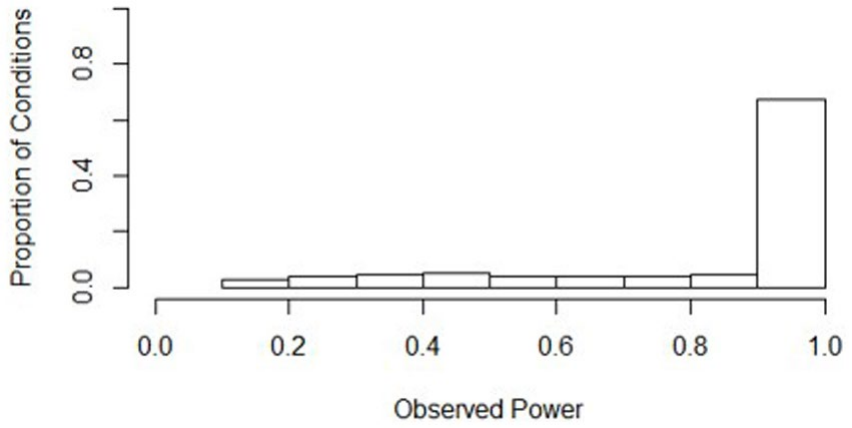
Distributions of power across all simulation conditions. Bins represent equal ranges of observed power in a simulation condition.

A histogram depicting the distribution of power is presented in Figure 1. Power was close to one for most conditions (mean = 0.841, *s* = 0.256). In fact, 72% of the 648 conditions had an average power above 0.8, which is considered appropriate power (Cohen, 1988). The lowest power observed was 0.128, meaning that less than 13% of the time, the slopes difference test was statistically significant when an effect should have been found across all replications for this condition. This lowest-powered condition, 436, had the lowest waves (3), the lowest class size (10), the lowest number of classrooms (20), an equal split of treatment and control classrooms, the smallest effect size (0.1), and the largest ICC (0.2). Being the lowest-powered condition, condition 436 had a similar set of parameters to other conditions where the slopes difference test had low power.

#### 3.1.2. ANOVA and *post-hoc* results for power

**Figure 2:**
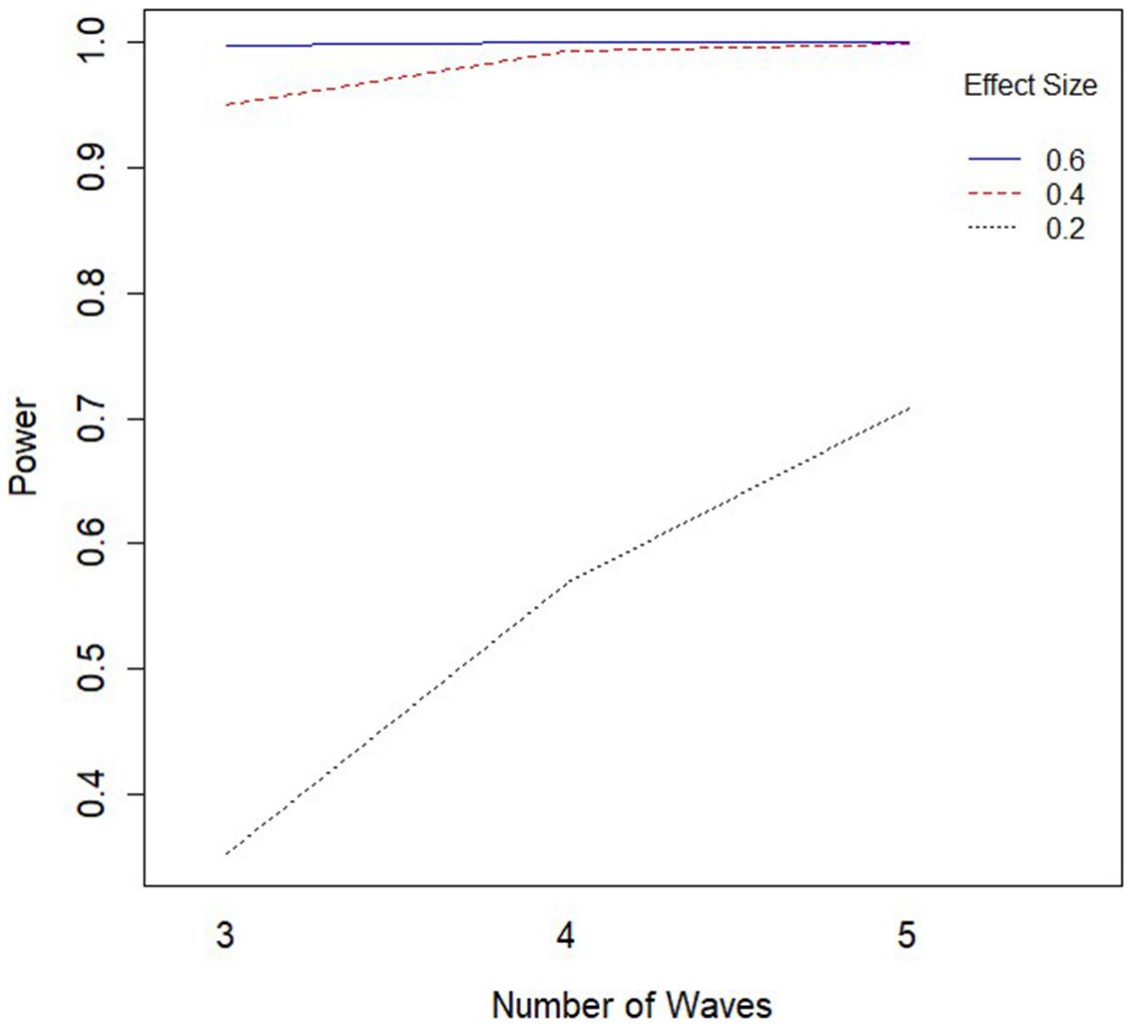
Interaction plot for number of waves and effect size predicting power. Effect size values of 0.6, 0.4, and 0.2 correspond to large, medium, and small effect sizes respectively; similar patterns were found for both class size and number of classrooms (replacing number of waves in this figure).

An ANOVA was conducted with power as the outcome and simulation conditions and their bivariate interactions as independent variables. Test results as well as effect sizes ( $\eta^{2}$ ) for the significant effect are summarized in Table 1. Starting with significant interaction effects, the only sizeable bivariate interactions were those that included effect size. Two of these interactions explained over 6% of the variance in power (number of waves * effect size and class size * effect size) and one interaction explained almost 4% (number of classrooms * effect size). These findings suggest that depending on the effect size for condition, the relationship between power and sample size-related variables changes. Since all the patterns were similar across all three sample-size related variables, the pattern can be seen in the provided plot for the interaction between the number of waves and the effect size predicting power (Figure 2). Figure 2 demonstrates that each of the sample size-related design factors (number of waves, size of class, and number of classrooms) had a stronger influence on power when the effect size is “small” (i.e., 0.2). Thus, these sample size-related variables had little to no influence on power when the effect size is “medium” (0.4) or “large” (0.6). All other interactions effects were non-significant.

**Table 1:** ANOVA results for simulation factors impacting power for the slopes difference test.

Type of effect	Factor	*F*	df	*p*	$\eta^{2}$
Main	Number of waves	803.913*	2	<0.001	**0.048**
	Size of class	809.524*	2	<0.001	**0.048**
	Number of classrooms	343.182*	3	<0.001	**0.031**
	Proportion in treatment	74.721*	1	<0.001	0.002
	Effect size	11433.155*	2	<0.001	**0.684**
	ICC	0.018	2	0.982	<0.001
Interaction	Waves: class	0.569	4	0.685	
	Waves: classrooms	0.394	6	0.883	
	Waves: treatment	0.360	2	0.698	
	Waves: effect	531.712*	4	<0.001	**0.064**
	Waves: ICC	0.048	4	0.996	
	Class: classrooms	0.366	6	0.900	
	Class: treatment	0.102	2	0.903	
	Class: effect	535.990*	4	<0.001	**0.064**
	Class: ICC	0.013	4	1.000	
	Classrooms: treatment	0.392	3	0.759	
	Classrooms: effect	212.300*	6	<0.001	**0.038**
	Classrooms: ICC	0.082	6	0.998	
	Treatment: effect	41.238*	2	<0.001	0.002
	Treatment: ICC	0.135	2	0.135	
	Effect: ICC	0.022	4	0.999	

*Indicates statistically significant at the 0.05 level; $\eta^{2}$ represents the proportion of the total variance in the DV that can be explained by the factor; only $\eta^{2}$ for statistically significant interaction effects shown; residual df = 576; a bold $\eta^{2}$ represents an effect size above the cutoff of 0.03.

Now considering main effects, the largest main effect was that of effect size ( $\eta^{2}=.684$ ) with more than 68% of the variance in power explained by effect size. Far behind effect size were number of waves, size of class, and number of classrooms, each explaining over 3% of the variance in power. However, the effect from these sample size-related factors on power depends primarily on whether the effect size is small. As such, their effects appear to have little influence unless effect size is small. Of the remaining factors, the proportion assigned to the treatment group and the ICC were not found to be influential in determining the power of the slopes difference test.

**Figure 3:**
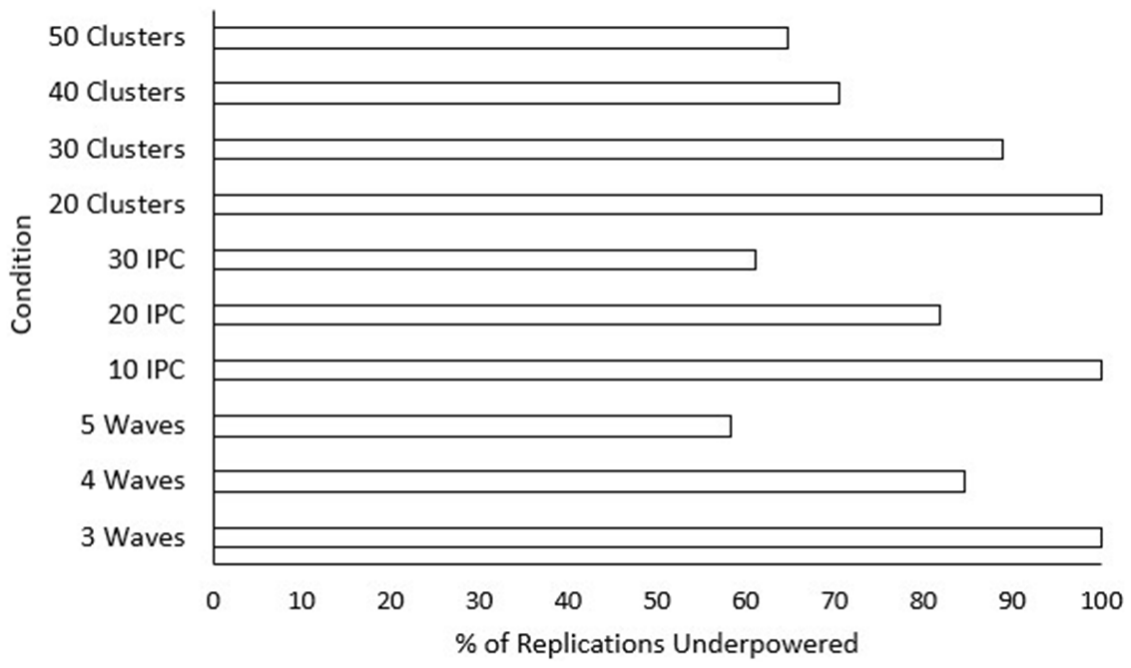
Percentage of replications underpowered across sample size conditions with a small effect size. IPC, individuals per cluster. A lower percentage is preferable for researchers.

**Figure 4:**
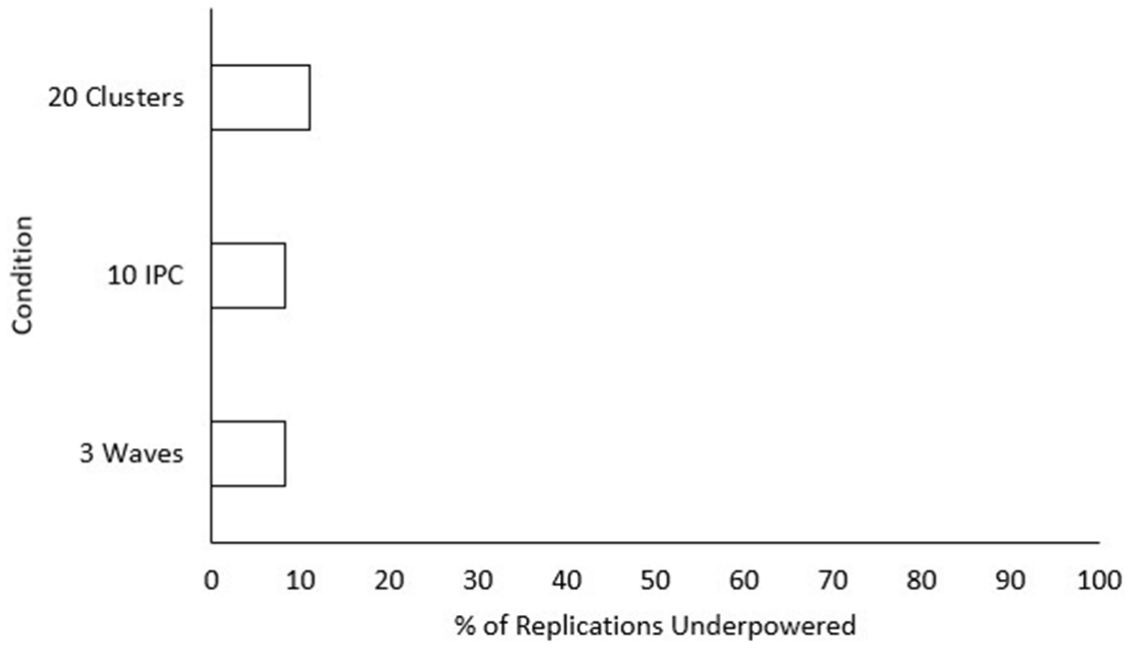
Percentage of replications underpowered across sample size conditions with a medium effect size. IPC, individuals per cluster. A lower percentage is preferable for researchers. Excluded conditions were 0% underpowered.

*Post-hoc* tests using Tukey’s HSD were conducted for factors where $\eta^{2}$ was larger than 0.03, in the order from largest to smallest $\eta^{2}$ . Only main effects were tested since pairwise differences for significant interaction conditions can be seen in Figure 2. The smallest effect size condition was found to have significantly lower power than both the “medium” and “large” effect size conditions. Power increased significantly for every increase in both number of waves and class size. Additionally, power was significantly higher for every increase in the number of classrooms included, aside from going from “40” classrooms to “50” classrooms. To assist practitioners, we have also included a chart displaying percentages of underpowered conditions (power < 0.8) across sample size conditions for small effect sizes (see Figure 3) and medium effect sizes (see Figure 4). Some medium effect size conditions and all large effect size conditions showed no underpowered replications, so they were not included in the figures.

### 3.2. Singularity and convergence rates for the slopes difference test

Supplemental to the analysis of power of the slopes difference test is how often the model had proper fit for each of the conditions. Singularity rate and convergence rate were examined similarly to power to determine the prevalence of these warnings as well as what conditions were more likely to lead to these outcomes. ANOVAs for both models only included main effects since this analysis is supplementary to the research questions. Effect sizes ( $\eta^{2}$ ) and Tukey’s HSD were calculated as well to assist the interpretation of the results.

#### 3.2.1. Descriptive statistics for singularity and convergence rates

**Figure 5:**
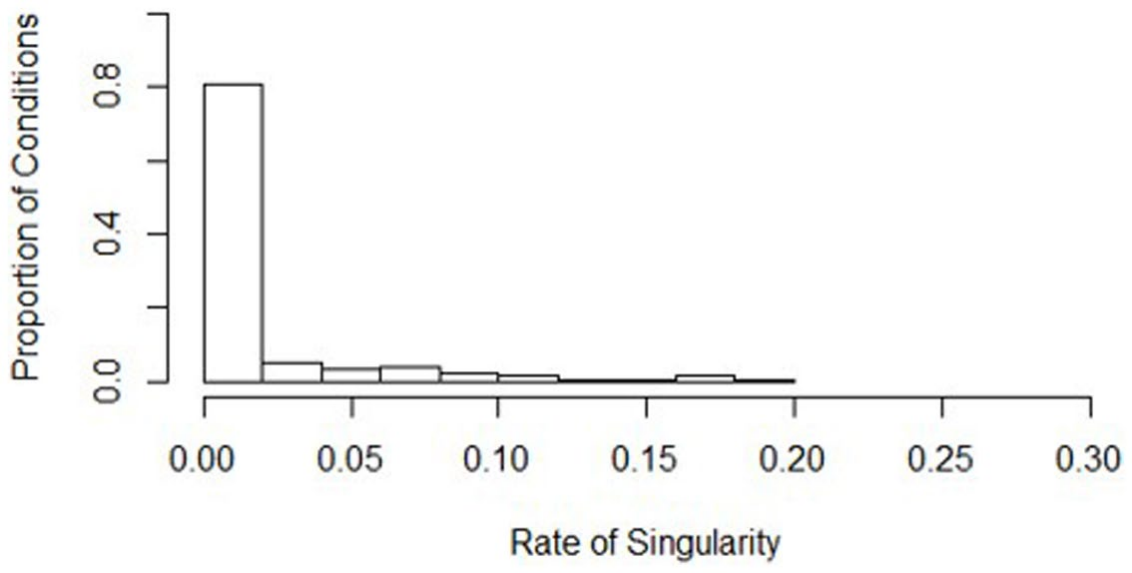
Distribution of singularity rates across all simulation conditions. Bins represent equal ranges of singularity rates in a simulation condition.

**Figure 6:**
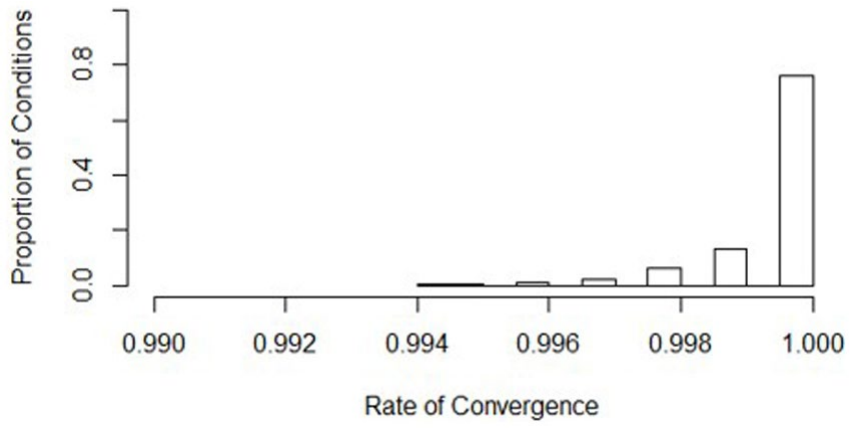
Distribution of convergence rates across all simulation conditions. Bins represent equal ranges of convergence rates in a simulation condition.

Non-convergence and singularity across the conditions were generally rare, with a high average convergence rate (mean = 0.999, *s* = 0.001) and a low average singularity rate (mean = 0.016, *s* = 0.035). Histograms show where the distributions of the conditions align (see Figures 5, 6). Values of the worst rates for convergence and singularity were 0.994 (condition 438) and 0.189 (condition 7), respectively. The lowest convergence condition had all the same conditions as the lowest powered condition (436), except for the effect size was large (0.5). Although this was the lowest convergence rate, the rate at which the model converged was still considerably high at 0.994. The lowest singularity rate condition also had mostly similar design conditions as the lowest powered condition (436), except for the ICC was low at 0.086. Overall, the slopes difference test did not have serious issues with singularity or non-convergence.

#### 3.2.2. ANOVA and *post-hoc* results for singularity and convergence rates

ANOVA Results for both convergence and singularity outcomes can be seen in Table 2. For convergence rate, the number of waves had the largest impact, explaining over 25% variance. The size of the class and the number of classrooms were also found to have relatively smaller effects, explaining 6.4 and 4.9% variance, respectively. This was also the only model where ICC came close to explaining 1% of the variance in the dependent variable ( $\eta^{2}$ = 0.009). *Post-hoc* tests (using *Tukey’s HSD*) were conducted for pairwise comparisons of factors where $\eta^{2}>0.03$ . Convergence rates were significantly lower for conditions where there were “three” waves, the class size was “10,” or there were “20” classrooms.

**Table 2:** ANOVA results for simulation factors impacting convergence and singularity rates for the overall model.

DV	Factor	*F*	df	*p*	$\eta^{2}$
Convergence	Number of waves	127.879*	2	<0.001	**0.251**
	Size of class	32.444*	2	<0.001	**0.064**
	Number of classrooms	16.471*	3	<0.001	**0.049**
	Proportion in treatment	3.055	1	0.081	
	Effect size	0.773	2	0.462	
	ICC	4.343*	2	0.013	0.009
Singularity	Number of waves	292.147*	2	<0.001	**0.383**
	Size of class	96.992*	2	<0.001	**0.127**
	Number of classrooms	37.315*	3	<0.001	**0.073**
	Proportion in treatment	0.004	1	0.948	
	Effect size	0.013	2	0.987	
	ICC	0.012	2	0.988	

*Indicates statistically significant at the 0.05 level; $\eta^{2}$ represents the proportion of the total variance in the DV that can be explained by the factor; only $\eta^{2}$ for significant results are shown; residual df = 635; a bold $\eta^{2}$ represents an effect size above the cutoff of 0.03.

**Figure 7:**
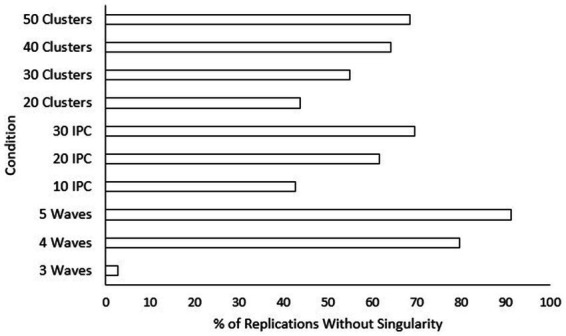
Percentage of replications without singularity across sample size conditions. IPC, individuals per cluster. A higher percentage is preferable for researchers.

**Figure 8:**
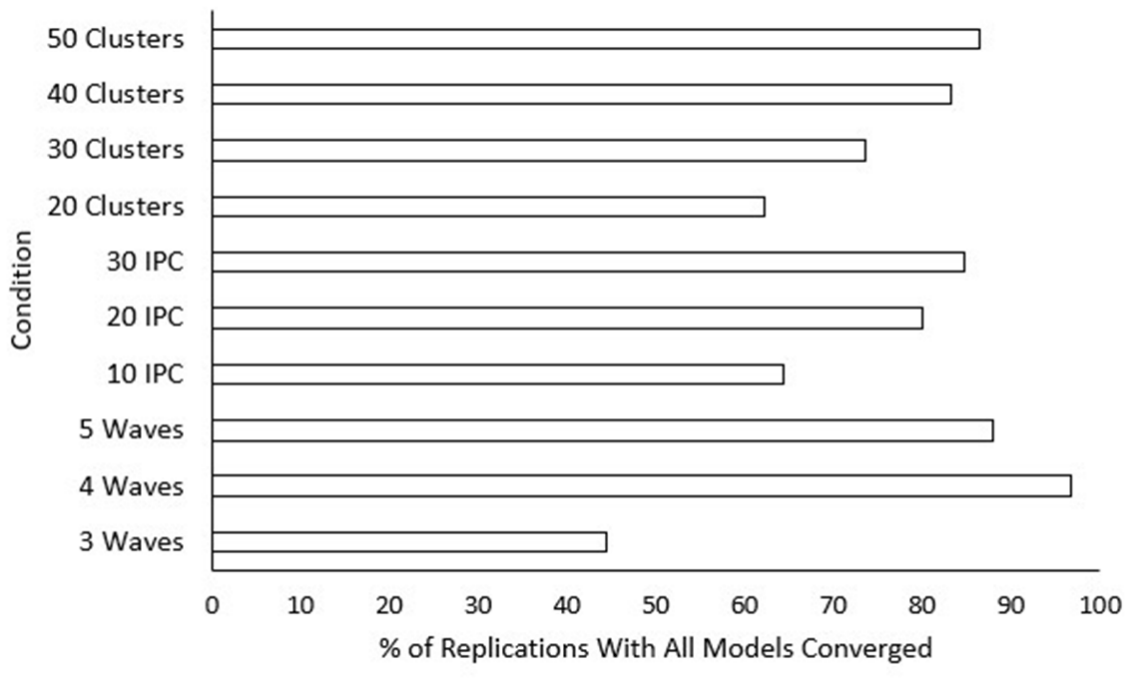
Percentage of replications with all models converged across sample size conditions. IPC, individuals per cluster. A higher percentage is preferable for researchers.

As shown in Table 2, three conditions explained sizeable portions of the variance in singularity. These effects once again came from factors related to sample size, with number of waves being large ( $\eta^{2}=0.383$ ), size of class medium ( $\eta^{2}=0.127$ ), and number of classrooms somewhat smaller ( $\eta^{2}=0.073$ ). *Post hoc* tests were also conducted for the current model. Rates of singularity were significantly higher for the “three-wave” group, and “20” classrooms condition. Singularity rates decreased significantly as class size condition increased. Additionally, “30” classrooms showed significantly higher singularity rates than “50” classrooms. To assist practitioners, we have also included figures displaying percentages of replications with no singularities (see Figure 7) and where all models converged (see Figure 8) across sample size conditions.

## 4. Discussion

The simulation and analyses conducted in this study were successful in answering the research questions as well as shedding light on the viability of these types of research designs and analyses. Following the pattern of the results section, the results of the power for the slopes difference test will be discussed in relation to the two research questions: (1) what was the power of the slopes difference test in a longitudinal multilevel analytical framework and (2) what factors affected this power and how. The supplemental results on singularity and convergence rate are then discussed, followed by implications of the study results.

### 4.1. Overall power and influencing factors for the slopes difference test

Answers for both research questions were found by (1) calculating descriptive statistics across design conditions and then (2) conducting an ANOVA with power as the dependent variable using the design conditions and their bivariate interactions as factors. These results showed that not only is the slopes difference test appropriately powered for most research conditions, but also that the slopes difference test has consistently higher power for longitudinal multilevel modeling than cross-sectional multiple regressions (i.e., Dawson and Richter, 2006).

For the first research question, the simulation showed that the slopes difference test was, on average, appropriately powered (i.e., 0.841) across all conditions. Dawson and Richter (2006) found an average power of 0.558 in their conditions similar to those used in the current study. This confirms the notion by Preacher and Sterba (2019) that there is greater power when using longitudinal-CRT in a multilevel framework. Since the observed power increase in the current study could be the result of introducing longitudinal design, the slopes difference test should also be examined in a longitudinal multiple regression context to see if power improves over the findings of Dawson and Richter (2006).

The answer to the second research question helps us better understand what conditions researchers should aim to meet in order to achieve appropriate power to detect slope differences. Howell (2012) discusses how power, Type I error, effect size, and sample size all relate to and influence each other in a closed system. It is this system of relationships that likely drove which factors were predictive of power in the current study. Considering the main effects of conditions on the power of the slopes difference test, effect size was by far the largest determining factor. In this study, the effect size of the slope difference indicated the difference between the slope for the low-aptitude control group and the low-aptitude treatment group. Finding a small effect means that the difference between the slopes for these two groups was about 0.1. In the current study, power levels in conditions where a small effect size existed were more than 0.43 lower than those under both the medium and large effect size conditions. This means that if all other conditions are the same but the effect size is small rather than medium or large, the slopes difference test is highly likely to be underpowered. These findings coincide with Dawson and Richter’s (2006) simulation of the slopes difference test in a cross-sectional multiple regression context. They found that unless the sample size was very large (i.e., 500), the power of the slopes difference test to detect small effects was highly underpowered. Sample sizes of 200 and 100 were required for properly powered slopes difference tests examining medium and large effects, respectively (Dawson and Richter, 2006).

Other sample size related design factors like number of waves, size of class, and number of classrooms were not nearly as influential as effect size but may still be considered as impactful. Specifically, power significantly increased when both the number of waves and the size of the class increased. These findings are consistent with Howell (2012) who said that effect size and sample size-related factors are the most influential for power. It would be interesting to have included even higher wave conditions and class sizes to see if the significant improvement in power provided by adding more tapers off at a certain level. However, for practical reasons using a larger number of waves or finding larger class sizes may not be realistic. Regarding the number of classrooms to collect data for, each increase in number led to a significant increase in power, although there was no significant increase in power going from 40 to 50 classrooms. This suggests that regardless of the values of other factors, once data from 40 classrooms have been collected, there is no added benefit for power of the slopes difference test in recruiting more classrooms. According to Hoyle and Gottfredson (2015), who studied cross-sectional applications of multilevel modeling, estimates from multilevel models are trustworthy with 10 or more clusters or with less than 10 clusters and a cluster size of 30 or more. The current study, while not disputing the suggestions of Hoyle and Gottfredson (2015), shows that power would significantly benefit from having much larger cluster size and numbers of clusters than they suggested. Although this may seem obvious to the statistician, practitioners who are less statistically savvy should now be warned: you are significantly less likely to find effects with the slopes difference test when meeting only the minimum sample sizes at all three levels explored in the current study (i.e., 3 waves, class size of 10, and 20 classrooms).

The bivariate interaction effects for design factors follow a similar pattern. That is, all the condition interactions that might be considered meaningful involved effect size as a moderator. This means that depending on what the effect size of the condition was, the influence of the other condition parameters on power for the slopes difference test changed. Specifically, conditions with the “small” effect size parameter showed an increased relationship between other factors and power. This finding corresponds with Scherbaum and Ferreter (2009) who state that statistical power in multilevel models can remain properly powered with much smaller samples when the observed effect size is medium or large. This conclusion was based on a statistical significance test for the group-level covariate (i.e., the omnibus interaction effect). The current study shows that this moderating effect of effect size on power also holds true for probing an interaction with the slopes difference test. The three interaction pairs of interest for the current study, number of waves with effect size, class size with effect size, and number of classrooms with effect size, all explain the variance of power between 3.8 and 6.4%. To illustrate, let us take one of these pairs. For the class size and effect size pair, the interaction effect shows that class size may become more important when the effect size is small and less important when effect size is large. For those hoping to gain greater power to detect small effect sizes, therefore, the number of waves, size of the class, and number of classrooms become especially important. In short, increasing each of these three design factors is the best way to increase the chances of detecting a small slope difference. Discussing the topic of sample size at different levels of a multilevel model, Snijders (2005) says that studies including more schools tend to be more expensive. Increasing the number of students can also be limited by the size of a school or classroom. Since the effect of sample size at all three levels on power is similar, the most prudent approach to increasing power would be to increase the number of waves.

It is also noteworthy to consider why changes in ICC were shown to have a minimal impact on power for the slopes difference test. Hox et al. (2010) who examined multivariate multilevel models, found that ICC had no effect on the accuracy of parameter estimates, even for between-group effects. According to Raudenbush (1997) medium (0.1) and large (0.2 or 0.5) ICCs require substantially smaller lower-level sample sizes (i.e., cluster size) to retain a power of 0.5 (0.8 being considered appropriate) for parameter estimates than a small ICC (0.05 or even 0.01). As such, the ICCs used in the current study may not have included small enough ICC conditions to demonstrate the main or moderating effect of ICC. Considering that a longitudinal-CRT design introduces greater potential for variance at the cluster level by assigning classrooms to treatment or control groups, the ICC conditions used for the current study are more likely to reflect a real-world research scenario than a small ICC category like 0.01. Therefore, the results from the current study are trustworthy for a longitudinal-CRT design.

### 4.2. Singularity and non-convergence: are they an issue?

Each of the 648,000 simulations conducted represents one random research study that an educational researcher might conduct. If the model does not converge or has singular fit for a particular study, the intended analysis cannot be conducted, and the research questions for the study would become more difficult to answer. Considering the complexity of a longitudinal-CRT and the multilevel model used, it is encouraging to see the high rates of convergence and low rates of singularity across all the simulation design conditions. Much like the way many people trust hand sanitizer to keep them sanitized when the bottle claims to kill 99.9% of germs, the longitudinal-CRT multilevel model can be trusted to converge over 99.9% of the time when one collects data that align with the conditions simulated in this study.

Although non-convergence and singularity are rare for longitudinal-CRT models, the results of this study allow researchers to figure out how many classrooms to include or whether they need to assign treatment groups equally to avoid non-convergence or singular fit. For both singularity and convergence, it is especially important to ensure having the appropriate number of waves. As illustrated, a three-wave design led to significantly lower convergence rates and significantly higher singularity rates than both four- and five-wave designs. This finding confirms prior assumptions by Chen et al. (2010) that four-wave designs are more reliable than three-wave designs and that a five-wave design may not be necessary because it does not significantly improve convergence and singularity rates.

Class size and number of classrooms are, although less strong than effect size or number of waves, informative for researchers trying to avoid non-convergence or singularity. Between class size and number of classrooms, class size was more important for reducing the likelihood of non-convergence and singularity. Although this goes against typical recommendations for multilevel modeling where increasing the number of classrooms would have more influence, the current result may be due to the model only having treatment as a covariate at the highest level of the model. Singularity rates went down with every increase in class size. Also, a class size of 10 was the only condition shown to have significantly worse convergence rates. Thus, applications of these types of models may be less likely to succeed in special education contexts. However, convergence and singularity rates are still reasonable even in conditions with only 10 students per classroom. The number of classrooms needed to help avoid non-convergence and singularity is 30 or greater. Conditions where only 20 classrooms were included performed significantly worse in terms of convergence and singularity. The results also showed that if researchers want to collect data from more than 30 classrooms to improve convergence and singularity rates, they need to go up to 50 classrooms to find any significant change. In a study comparing lower-level (e.g., students) and group-level (e.g., schools) sample sizes within a multilevel context, Maas and Hox (2005) found that only group-level sample sizes were influential for convergence rates. However, Maas and Hox (2005) examined conditions where convergence got as low as 90.1%. Additionally, the design used by Maas and Hox (2005) was cross-sectional whereas the current study was longitudinal, so the within-group sample size for the current study includes both waves and class size. Being that the current study used a longitudinal model where within-group sample sizes (i.e., the class size and number of waves) and convergence rates far exceeded those examined by Maas and Hox (2005), the influence of sample size (i.e., number of waves, class size, and number of classrooms) on power was likely more balanced between levels of the analysis. It should also be noted that differences for convergence rates in the current study were so small that they should be considered inconsequential for research in practice.

### 4.3. Implications, limitations, and conclusion

Educational research positively impacts educational structures and enhances student learning (Reddy, 2016). How much more informative and meaningful could educational research be if statistical techniques that more accurately reflect the context of the data are used? Advances in technology have opened the door for precision education researchers to use advanced techniques like longitudinal multilevel modeling that accurately reflect the data structure they are studying. Although scarcely utilized, probing techniques like the slopes difference test are conceptually a good fit for ATI or STI frameworks and should be utilized by precision education researchers more. In fact, the results of the current study show that the slopes difference test is more viable in a longitudinal-CRT within a multilevel framework than in its original context of single level cross-sectional multiple regression. To better understand differences between treatments on the educational outcomes for students of different aptitudes or skills, researchers should consider a slopes difference test as a powerful tool when using a longitudinal-CRT design. Based on the findings of the current study as well as prior research (i.e., Dawson and Richter, 2006), the slopes difference test is recommended for examining medium or large effects for both multiple regression and longitudinal multilevel models. Researchers may still use the slopes difference test for a small effect size scenario, but the test may be underpowered. Additionally, to improve power and reduce chances of non-convergence or singularity, researchers should first increase the number of waves, then the size of the class, and finally the number of classrooms. Taken in this order, these improvements are also the most cost-effective way to improve a longitudinal-CRT study. Although this recommendation is somewhat contrary to that typical in multilevel modeling, the model for the current study, where the only cluster-level covariate is treatment and the model is longitudinal, lends itself to being less influenced by number of clusters. This combined with the findings of the current study, where number of waves had a substantially larger influence on convergence and singularity rates, leads us to conclude that the number of waves is the most impactful factor for improving results for a slopes difference test in a longitudinal-CRT.

One thing to consider when discussing the applicability of the current results in the context of major interest (ATI/STI research in precision education) is the fact that many studies in this context have historically utilized a pre-post design rather than the more powerful longitudinal design with at least 3 waves where multilevel models can be utilized (see Spybrook and Raudenbush, 2009; Wolfe et al., 2009; Clements et al., 2011; Fuchs et al., 2014). The benefits of designing research studies that include multiple waves of longitudinal data have been discussed at length in this paper. Given that the current study has shown that a slopes difference test is effective for probing ATI and STI effects, especially for longitudinal CRT designs, it is our recommendation that future researchers collect more waves of data, thus allowing for more powerful and informative statistical analyses. Due to the complex nature of the research design and statistical tests discussed here, work should be done to educate researchers in the field of precision education on how to design and analyze studies such that powerful tests like the slopes difference test can be utilized.

To improve upon the current study, more simulation conditions could be considered to more fully understand how power can be influenced for the slopes difference test. For example, smaller ICC conditions could be considered to confirm whether a small ICC would affect power for the slopes difference test. Additionally, larger sample size conditions (e.g., number of waves and class size) could be included to determine if the positive effects of increased sample size eventually become insignificant. While these additional conditions do not fit well with a precision education context where a longitudinal-CRT design is used, other areas of research may need to consider data with these conditions. Education researchers might argue that the effect size conditions represented here (in accordance with Cohen, 1988) are not consistent with common effect sizes in educational intervention studies (Kraft, 2020). It would be beneficial to expand the current effect size conditions to account for these discrepancies in future research.

It can also be noted that the model framework examined here represents a simpler one than many researchers would utilize. For example, many longitudinal CRT studies also consider covariates at any level of the model. In general, introducing multiple predictors can add to model complexity, thereby increasing the risk of convergence issues or singularity. Additional issues such as reduced statistical power and multicollinearity could lead the researcher to miss or even misinterpret model effects (see Shieh and Fouladi, 2003). Many researchers fall prey to the allure of including “statistical controls” in their models assuming it will appropriately adjust the effects of interest (such as an ATI). However, there is a body of work suggesting that such practices not only detract from potential findings but can also lead to erroneous conclusions for variable relationships of interest (see Spector and Brannick, 2011; Carlson and Wu, 2012). As such, we decided to utilize the most parsimonious model for examining ATIs to avoid these issues. Further research should be done to determine the impacts of the inclusion of covariates in the current model. Regardless of these limitations, the current study serves as a starting point for understanding the utility of the slopes difference test in a longitudinal-CRT design.

Findings from the current study may also be generalized to research in other fields utilizing similar parameters in a longitudinal CRT context. For example, medical researchers who often compare treatment effects among several randomly assigned physicians could benefit from the slopes difference test, especially if they wish to consider how patient characteristics might impact treatment effectiveness on patient outcomes. One study that could stand to benefit from a slopes difference test is provided in Grandes et al. (2011). This study evaluated the effectiveness of a physical activity promotion intervention on patients by utilizing multilevel modeling and a longitudinal CRT. One of the effects the authors were interested in was whether the prescriptions given to the patient moderated the intervention’s effectiveness on the various outcomes examined in the study. Given that the study examined 4 waves, 56 clusters, and an average of approximately 65 individuals per cluster, the data would be a good candidate for conducting a successful slopes difference test given the results of the current study. Other fields outside of ATI and STI research stand to benefit from the results of the current study such as: education (see Rosário et al., 2020), psychology/psychiatry (see Brathwaite et al., 2022), and business (see Janssens et al., 2020).

The current study has extended the slopes difference test to longitudinal multilevel modeling and shown that it is a powerful tool for discovering differences within a precision education context where longitudinal-CRT and multilevel modeling are applied. In addition, we have made evidence-based recommendations for how to improve the power of the slopes difference test and avoid modeling errors such as non-convergence and singularity. The hope is that this study will lead to increased use of the slopes difference test in a longitudinal-CRT design and thus benefit research design in precision education and related areas.

## Data availability statement

The datasets presented in this study can be found in online repositories. The names of the repository/repositories and accession number(s) can be found in the article/supplementary material.

## Author contributions

TD conducted the literature review, designed the methods, conducted the analysis, and wrote the manuscript. QC had an advisory role throughout the research process and made major contributions to the development and refinement of research questions, design of the study, model specifications, and discussion of the results from the study. QC was also involved in the writing process by editing, organizing, and polishing the whole manuscript. All authors contributed to the article and approved the submitted version.

## Conflict of interest

The authors declare that the research was conducted in the absence of any commercial or financial relationships that could be construed as a potential conflict of interest.

## Publisher’s note

All claims expressed in this article are solely those of the authors and do not necessarily represent those of their affiliated organizations, or those of the publisher, the editors and the reviewers. Any product that may be evaluated in this article, or claim that may be made by its manufacturer, is not guaranteed or endorsed by the publisher.
